# Origins of a cyanobacterial 6-phosphogluconate dehydrogenase in plastid-lacking eukaryotes

**DOI:** 10.1186/1471-2148-8-151

**Published:** 2008-05-17

**Authors:** Shinichiro Maruyama, Kazuharu Misawa, Mineo Iseki, Masakatsu Watanabe, Hisayoshi Nozaki

**Affiliations:** 1 Department of Biological Sciences, Graduate School of Science, University of Tokyo, 7-3-1 Hongo, Bunkyo, Tokyo 113-0033, Japan; 2 Hayama Center for Advanced Studies, Graduate University for Advanced Studies (SOKENDAI), Hayama, Kanagawa 240-0193, Japan; 3 School of Advanced Sciences, Graduate University for Advanced Studies (SOKENDAI), Hayama, Kanagawa 240-0193, Japan; 4 Research Program for Computational Science, Riken, 4-6-1 Shirokane-dai, Minato-ku, Tokyo 108-8639, Japan

## Abstract

**Background:**

Plastids have inherited their own genomes from a single cyanobacterial ancestor, but the majority of cyanobacterial genes, once retained in the ancestral plastid genome, have been lost or transferred into the eukaryotic host nuclear genome via endosymbiotic gene transfer. Although previous studies showed that cyanobacterial *gnd *genes, which encode 6-phosphogluconate dehydrogenase, are present in several plastid-lacking protists as well as primary and secondary plastid-containing phototrophic eukaryotes, the evolutionary paths of these genes remain elusive.

**Results:**

Here we show an extended phylogenetic analysis including novel *gnd *gene sequences from Excavata and Glaucophyta. Our analysis demonstrated the patchy distribution of the excavate genes in the *gnd *gene phylogeny. The *Diplonema *gene was related to cytosol-type genes in red algae and Opisthokonta, while heterolobosean genes occupied basal phylogenetic positions with plastid-type red algal genes within the monophyletic eukaryotic group that is sister to cyanobacterial genes. Statistical tests based on exhaustive maximum likelihood analyses strongly rejected that heterolobosean *gnd *genes were derived from a secondary plastid of green lineage. In addition, the cyanobacterial *gnd *genes from phototrophic and phagotrophic species in Euglenida were robustly monophyletic with Stramenopiles, and this monophyletic clade was moderately separated from those of red algae. These data suggest that these secondary phototrophic groups might have acquired the cyanobacterial genes independently of secondary endosymbioses.

**Conclusion:**

We propose an evolutionary scenario in which plastid-lacking Excavata acquired cyanobacterial *gnd *genes via eukaryote-to-eukaryote lateral gene transfer or primary endosymbiotic gene transfer early in eukaryotic evolution, and then lost either their pre-existing or cyanobacterial gene.

## Background

A cyanobacterium-like ancestor gave rise via primary endosymbiosis to a distinctive endosymbiotic organelle, the plastid (primary plastid), in eukaryotic cells [1,2]. Some eukaryotic lineages retained the plastid through successive generations, and its photosynthetic ability enabled them to grow autotrophically. Some may have lost the plastid, and returned to their previous heterotrophic state, whereas others may have never experienced such an endosymbiotic event.

Green plants (green algae and land plants), Glaucophyta and red algae are primary plastid-containing photosynthetic eukaryotes. They are classified into a single super-group, Archaeplastida, among the six 'super-groups' proposed by Adl et al. [3]. It is generally believed that the majority of the cyanobacterial genes (genes sharing their origins with cyanobacterial homologues) found in the nuclear genomes of extant Archaeplastida were recruited from cyanobacterium-like endosymbionts via endosymbiotic gene transfer (EGT) [4-6].

Other algae in several independent lineages are thought to have secondarily acquired plastids by engulfing primary photosynthetic eukaryotes. These have evolved into secondary plastid-containing photosynthetic eukaryotes (secondary phototrophs) [1,2]. Most secondary plastids in the super-group Chromalveolata, which consists of Stramenopiles, Alveolata, Haptophyta and Cryptophyta, are derived from red algae. Chlorarachniophyta in the Rhizaria group and Euglenida in the Excavata group possess secondary plastids derived from green algal ancestors [7-9]. A large number of plastid-related cyanobacterial genes were further introduced into nuclear genomes of secondary phototrophs via secondary EGT [10-12].

Although several studies have reported cyanobacterial genes in plastid-lacking eukaryotes [13,14], *gnd *genes are remarkable in their broad distribution among primary and secondary plastid-containing photosynthetic eukaryotes as well as among plastid-lacking protists [15,16]. The *gnd *gene encodes an oxidative pentose phosphate pathway enzyme, 6-phosphogluconate dehydrogenase, which is important in regulating sugar metabolism and intracellular redox state. Previous studies reported that the *gnd *gene is widely conserved among eukaryotes and eubacteria [17], and showed that there are two types of *gnd *genes; one is phylogenetically close to cyanobacterial *gnd *genes (termed 'cyanobacterial *gnd*'), and the other resembles cytosol-localized *gnd *genes in Opisthokonta (termed 'eukaryotic ancestral *gnd*'). Cyanobacterial *gnd *genes are present not only in primary and secondary phototrophs, but also in plastid-lacking protists. These include the plant pathogen *Phytophthora *that is classified into the super-group Chromalveolata, and the heterolobosean amoebo-flagellates that are classified into the super-group Excavata [15,16]. These pioneering studies suggested a possible scenario that cyanobacterial *gnd *genes were introduced via primary or secondary endosymbiosis [15-17]. Nevertheless, the origin and evolutionary relationships of these genes in photosynthetic and plastid-lacking eukaryotes remains inconclusive.

We present here an extended analysis of the phylogeny of *gnd *genes with emphasis on the plastid-lacking excavate protists. We also discuss the origin and evolutionary history of the cyanobacterial genes in plastid-lacking protists, within the scope of previously proposed hypotheses on ancient lateral gene transfer (LGT) and EGT events.

## Methods

### Culture material

*Diplonema papillatum *(ATCC No. 50162) was axenically cultured at 25°C in artificial seawater supplemented with 1% horse serum (Invitrogen, Carlsbad, CA, USA), 1 × Daigo IMK medium (Nippon Pharmaceutical, Tokyo, Japan) and 0.1% tryptone. *Peranema trichophorum *cells, co-cultured with *Chlorogonium *sp., were provided by Dr. Toshinobu Suzaki (Kobe University). *Euglena gracilis *Z (NIES-48) was cultured as described previously [18].

### cDNA Library construction and PCR-based gene isolation

*D. papillatum *genomic DNA was extracted using the DNeasy plant mini kit (Qiagen, Hilden, Germany). *P. trichophorum *full-length cDNA sequences were synthesized using the SV total RNA isolation system (Promega, Madison, WI, USA) and the CapFishing full-length cDNA kit (Seegene, Seoul, Korea). Glaucophyte cDNAs (*Cyanophora paradoxa *NIES-547, *Gloeochaete wittrockiana *SAG 46.84 and *Cyanoptyche gloeocystis *SAG 34.90) were prepared as described in the previous study [19], and used as templates for gene isolation. Fragments of *gnd *genes were amplified using nested-degenerated primers based on the conserved amino acid motif GLAVMGQN for forward primers (GGIYTIGCIGTIATGGGICA or YTIGCIGTIATGGGICARAA) and QAQRDFFG for reverse primers (CCRAARAARTCICKYTGIGC or AARAARTCICKYTGIGCYTG). PCR products and cDNA clones were sequenced directly or after TA-cloning, using an ABI PRISM 3100 genetic analyzer (Applied Biosystems, Foster City, CA, USA) with a BigDye Terminator Cycle Sequencing Ready Reaction kit v. 3.1 (Applied Biosystems). Expressed sequence tags (ESTs) of *Euglena gracilis *(3,934 sequenced clones, average length 532 bp) were generated by sequencing cDNA clones selected at random from a cDNA library (average insert size, >1 kbp) constructed using a cDNA synthesis kit (Stratagene, Cedar Creek, TX, USA). The EST sequencing was performed at the Dragon Genomics Center, Takara Bio Inc. (Yokkaichi, Japan). A clone harboring the full-length *gnd *gene sequence was identified by BLAST search.

### Phylogenetic analysis

The data matrix of *gnd *genes was based on the amino acid alignment in Andersson and Roger [15]. We excluded amitochondrial and/or parasitic eukaryotes, which might cause long branch attraction due to unusual nucleotide substitutions [15,20,21]. We included the novel sequences determined in this study (Table 1), and sequences identified by the BLAST program from the *Galdieria sulphuraria *genome database [22], the Joint Genome Institute [23] and the *Acanthamoeba castellanii *EST database in TBestDB [24]. The sequences were aligned using CLUSTAL X [25] and manually refined using SeaView [26]. The data matrix was made with 63 taxa and 437 amino acid sites (available upon request to SM). Data matrices excluding Heterolobosea (61 taxa, 437 sites) and including amitochondrial and/or parasitic eukaryotes (72 taxa, 437 sites) were also prepared to construct additional trees (Additional files 1 and 2, respectively).

**Table 1:** Sequences encompassing the EW signature and accession numbers of *gnd *genes identified in this study

Species name	Taxonomy	EW signature	Accession number
*Cyanophora paradoxa*	Glaucophyta	IDGGN**EW**YENTE	AB425331
*Gloeochaete wittrockiana*	Glaucophyta	IDGGN**EW**YKNTE	AB425332
*Cyanoptyche gloeocystis*	Glaucophyta	IDGGN**EW**YLNTE	AB425333
*Euglena gracilis*	Euglenida	VDGGN**EW**FPNSQ	AB425328
*Peranema trichophorum*	Euglenida	IDGGN**EW**FPNTL	AB425329
*Diplonema papillatum*	Diplonemea	IDGGNSHFPDSI	AB425330

EW signature residues conserved among cyanobacterial *gnd *genes [15] are indicated in bold.

Bayesian inference was performed with the program MrBayes version 3.1.2 [27] using the WAG matrix of amino acid replacements assuming a proportion of invariant positions and four gamma-distributed rates (WAG+I+Γ4 model). For the MrBayes consensus trees, 1,000,000 generations were completed with trees collected every 100 generations. One thousand replicates of bootstrap analyses by maximum likelihood (ML) method were performed using PhyML version 2.4.4 [28] with the WAG+I+Γ4 model on two SunFire 15K machines, each of which has 96 CPUs. Bootstrap values (1,000 replicates) based on maximum parsimony (MP) analysis were calculated with PAUP 4.0 b10 with TBR heuristic search [29]. For exhaustive ML analysis, topology-dependent sitewise likelihood values were calculated using TREE-PUZZLE version 5.2 under a WAG+F+Γ8 model [30]. Alternative tree topologies were analyzed with the approximately unbiased (AU) [31] and Kishino-Hasegawa (KH) [32] tests, and the resampling estimated log-likelihood (RELL) bootstrap support values [33], using the CONSEL package [31].

## Results and Discussion

### Phylogenetic and statistical analysis of *gnd *genes

**Figure 1:**
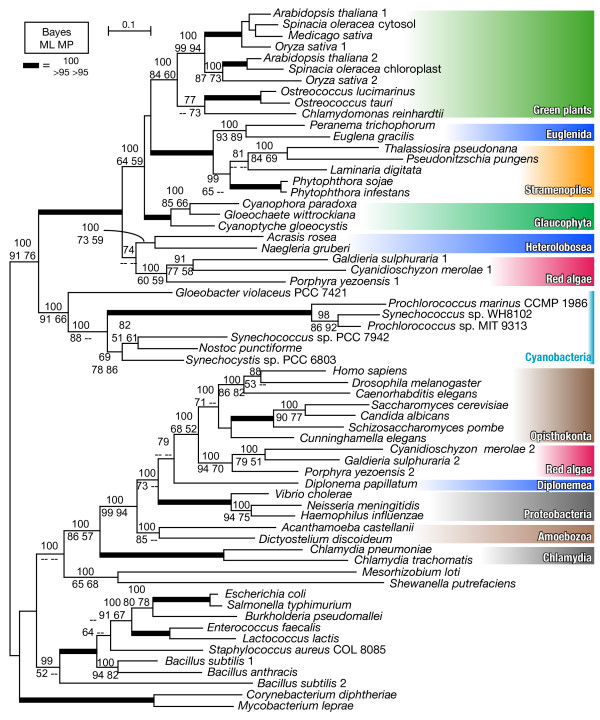
**MrBayes consensus tree of *gnd *genes, constructed with 437 amino acid sites from 63 taxa**. Bayesian posterior probabilities (Bayes) (70% or more) and maximum likelihood (ML) and maximum parsimony (MP) bootstrap support values (50% or more) are shown. The thick branches are represented as described in the figure.

Fig. 1 shows a Bayesian consensus tree from a matrix with 63 taxa, with Bayesian posterior probabilities (Bayes) of 70% or more, and ML and MP bootstrap support values of 50% or more. As reported previously [15,16], all the red algae examined have both cyanobacterial and eukaryotic ancestral *gnd *genes. Although several excavate *gnd *genes (Heterolobosea and Euglenida) were cyanobacterial in agreement with the previous studies [15,16], the *gnd *gene from another excavate species, *D. papillatum*, was found to group with Opisthokonta and red algal eukaryotic ancestral genes (Bayes|ML|MP = 79|--|--). Several proteobacterial species (*Vibrio, Neisseria *and *Haemophilus*) showed a weak affinity to eukaryotic genes (Bayes|ML|MP = 100|73|--), and Amoebozoa was located outermost in the eukaryotic ancestral clade (Bayes|ML|MP = 100|99|94). Notably, red algae and excavate genes shared basal positions within each of the cyanobacterial and eukaryotic ancestral clades. As shown in *Trypanosoma, Giardia *and *Trichomonas *[15], the EW sequence signature, which is unique to the cyanobacterial *gnd *genes, was absent in the *D. papillatum gnd *gene (Table 1, Additional file 3), confirming its non-cyanobacterial origin. However, the parasitic excavates were positioned outside of the eukaryotic ancestral clade with weak support values in the tree of 72 taxa (Additional file 2), possibly due to long branch attraction. Whether the genes from parasitic Excavata truly shared the same origin as known free-living Excavata genes, or were independently acquired via prokaryote-to-eukaryote LGT is open to further investigation of evolutionary signals and functional characterization. Our results and currently available genome information suggest that, while each red algal species possesses both cyanobacterial and eukaryotic ancestral genes and supposedly use them in different cellular compartments, free-living Excavata examined to date have just one or the other.

Cyanobacterial genes from bikonts [34] (namely Archaeplastida, Stramenopiles and Excavata in this study) were robustly monophyletic (Bayes|ML|MP = 100|100|98) and showed a strong affiliation with the genes from cyanobacteria (Bayes|ML|MP = 100|91|76) (Fig. 1). In the cyanobacterial gene clade, each of the three divisions of Archaeplastida (green plants, Glaucophyta and red algae) was monophyletic but separately located (Fig. 1). Glaucophyte *gnd *genes formed a monophyletic group with green plants, Euglenida, and Stramenopiles with moderate support values (Bayes|ML|MP = 100|64|59). Secondary phototrophs and the plastid-lacking heterotrophic relatives from Euglenida and Stramenopiles were robustly monophyletic (Bayes|ML|MP = 100|100|100). Plastid-lacking heterolobosean protists and red algae were located at the basal position in the cyanobacterial clade, weakly forming a monophyletic group (Bayes|ML|MP = 74|-|-).

To test the possibility that the plastid-lacking excavate protists acquired *gnd *genes via secondary endosymbiosis of a green alga [15], we carried out an exhaustive ML analysis for calculating the likelihood values of alternative tree topologies. First, based on the topology in Fig. 1, we defined six groups in which monophyly was confirmed by all three methods (Bayes = 100, ML > 50, MP > 50): green plants (Green), Glaucophyta (Glauco), Stramenopiles + Euglenida (EuSt), Heterolobosea (Htrl), red algae (Red) and others (Outgroup). Then, we constructed all possible 105 trees, fixing the intra-group topologies of the six monophyletic groups as in Fig. 1, and calculated probabilities of each tree for AU and KH tests (Table 2, Additional file 3). All possible 15 trees supporting the monophyly of Green + Htrl were rejected by both AU and KH tests at the 5% confidence level. All possible nine trees supporting monophyly of Green + EuSt + Htrl groups were also rejected by both tests at the 5% confidence level (Table 2).

**Table 2:** Comparison of alternative tree topologies by exhaustive maximum likelihood (ML) analysis

Tree^a^	Topology^b^	ΔlnL^c^	S.E.	pAU^d^	pKH^d^	RELL^e^
1	(Out, (Red, Htrl), (EuSt, (Glauco, Green)));	<-27143.41>	-	0.867	0.758	0.283
36	(Out, Red, ((Htrl, Green), (EuSt, Glauco)));	30.2	14.4	0.038	0.020	5.00E-06
49	(Out, ((Red, (Htrl, Green)), EuSt), Glauco);	54.7	17.8	0.013	0.003	2.00E-04
54	(Out, ((Red, (Htrl, Green)), Glauco), EuSt);	40.1	19.3	0.011	0.024	3.00E-04
56	(Out, Red, (((Htrl, Green), Glauco), EuSt));	27.8	15.8	0.009	0.038	4.00E-04
58	(Out, ((Red, Glauco), (Htrl, Green)), EuSt);	41.6	19.4	0.009	0.021	3.00E-06
70	(Out, ((Red, EuSt), (Htrl, Green)), Glauco);	52.8	18.2	0.004	0.005	3.00E-07
71	* (Out, (Red, (Htrl, (EuSt, Green))), Glauco);	48.9	15.5	0.004	0.002	1.00E-05
82	* (Out, (Red, ((Htrl, Green), EuSt)), Glauco);	57.1	17.8	0.001	0.002	3.00E-06
85	(Out, (Red, (EuSt, Glauco)), (Htrl, Green));	44.1	15.0	0.001	0.004	2.00E-05
86	(Out, (Red, ((Htrl, Green), Glauco)), EuSt);	39.2	19.1	5.00E-04	0.025	6.00E-06
87	* (Out, Red, (((Htrl, EuSt), Green), Glauco));	29.5	14.8	4.00E-04	0.025	1.00E-06
89	(Out, ((Red, EuSt), Glauco), (Htrl, Green));	51.3	18.0	2.00E-04	0.005	3.00E-06
91	* (Out, Red, ((Htrl, (EuSt, Green)), Glauco));	27.9	14.5	1.00E-04	0.028	1.00E-05
93	* (Out, (Red, Glauco), (Htrl, (EuSt, Green)));	50.0	16.3	1.00E-04	0.003	3.00E-06
94	* (Out, (Red, ((Htrl, EuSt), Green)), Glauco);	54.0	18.5	1.00E-04	0.004	1.00E-06
95	(Out, (Red, EuSt), ((Htrl, Green), Glauco));	44.8	18.5	6.00E-05	0.012	2.00E-06
96	(Out, (Red, (Htrl, Green)), (EuSt, Glauco));	41.4	15.0	3.00E-05	0.005	5.00E-07
98	* (Out, (Red, Glauco), ((Htrl, EuSt), Green));	52.5	18.7	2.00E-06	0.006	7.00E-07
101	(Out, ((Red, Glauco), EuSt), (Htrl, Green));	55.7	18.0	2.00E-40	0.003	2.00E-15
103	* (Out, (Red, Glauco), ((Htrl, Green), EuSt));	57.0	18.2	2.00E-53	0.003	1.00E-17
104	* (Out, Red, (((Htrl, Green), EuSt), Glauco));	32.8	15.0	3.00E-56	0.016	8.00E-19

^a^Best tree (tree 1) and trees supporting the monophyly of green plants + Heterolobosea among all the possible 105 trees retaining six monophyletic groups in Fig. 1.

^b^Green, green plants; Htrl, Heterolobosea; Glauco, Glaucophyta; EuSt, Euglenida and Stramenopiles; Red, red algae; Out, eukaryotic ancestral clade and cyanobacteria. Intra-group topologies of six groups are fixed as shown in Fig. 1.

^c^Difference in the log-likelihood value of alternative tree versus the 'best' tree.

^d^Probability values of the approximately unbiased (AU) and Kishino-Hasegawa (KH) tests.

^e^Bootstrap support value of resampling estimated log-likelihood with 10,000 replicates.

Asterisks indicate the topologies supporting the monophyly of Green + Htrl + EuSt.

Although our tree topology in Fig. 1 suggests that cyanobacterial genes from bikonts were originally acquired via a single gene transfer event from cyanobacteria, there are two possible explanations of their origin as discussed in the previous study [15]; early primary EGT from the ancestral plastid genome, or prokaryote-to-eukaryote LGT from a close relative of extant cyanobacteria independently of EGT. We favor the former scenario for the following reasons: 1) the *gnd *gene product is functionally plastid-related, and is enzymatically localized to the plastid in green plants [17]; and 2) the overall tree topology in Fig. 1 is consistent with a recent multigene phylogeny of eukaryotes based on slowly evolving nuclear genes [19].

### Origins of plastid-lacking excavate *gnd *genes

Heterolobosean *gnd *genes occupied the basal positions in the cyanobacterial clade and weakly formed a monophyletic group with red algae. Although our tree topology suggests that euglenid and heterolobosean *gnd *genes are distantly related, previous studies have not clearly excluded the single secondary-plastid origin of these genes [15,16]. To test whether the heterolobosean *gnd *genes could originate with secondary EGT as suggested by the 'plastids-early' hypothesis for secondary plastids in Euglenida [8], we verified the possibility that the cyanobacterial *gnd *genes in plastid-lacking heterolobosean protists and green plants could be potentially monophyletic, using confidence tests based on exhaustive ML analyses (Table 2). According to the plastids-early hypothesis for secondary plastids in Euglenida [8], the secondary endosymbiosis of green alga occurred in the common ancestor of Euglenida and Heterolobosea, and extant plastid-lacking protists within these taxa have secondarily lost their plastids and photosynthesis-related genes. Although this hypothesis is contentious [1,8], it is worth verifying because this is the leading explanation for the acquisition of cyanobacterial genes through secondary endosymbionts in Heterolobosea. Considering that the orientation of LGT between the ancestors of Stramenopiles and Euglenida is unknown, we examined two possibilities on the origin of the euglenid and heterolobosean *gnd *genes. First, we examined the possibility that ancient euglenid *gnd *was transferred into the common ancestor of Stramenopiles, which postulates the monophyly of Stramenopiles, Euglenida, Heterolobosea and green plants. Then we examined the second possibility that an ancient stramenopile *gnd *was acquired by the euglenid ancestor, which assumes that Heterolobosea and green plants are exclusively monophyletic. All the trees supporting first or second possibilities were rejected by AU and KH tests at the 5% confidence level (Table 2). These results suggested that heterolobosean *gnd *genes were not secondary green plastid-derived, and that the *gnd *gene phylogeny did not support the plastids-early hypothesis [8,35]. Taken together, our data disallowed the plastids-early hypothesis, and showed that a secondary endosymbiotic origin of the *gnd *genes from green alga into plastid-lacking excavate protists is unlikely.

It is striking that Euglenida is monophyletic with Stramenopiles in the cyanobacterial clade (Fig. 1). Recent phylogenetic analyses of the plastid-encoded and nuclear-encoded plastid-targeted genes suggest that the ancestor of euglenid secondary plastids branches within green algae, inconsistent with our *gnd *tree topology [9,36]. The monophyly of cyanobacterial *gnd *genes from *E. gracilis *and plastid-lacking *P. trichophorum *further suggests that euglenid *gnd *genes have not been recruited via secondary EGT of a green alga, because the 'plastids-recent' hypothesis argues that eukaryovorous euglenid species such as *P. trichophorum *diverged before the secondary endosymbiotic event in the Euglenida lineage [8]. Meanwhile, the presence of the cyanobacterial genes in Stramenopiles, including photosynthetic algae and the plastid-lacking oomycete *Phytophthora*, is apparently consistent with the 'Chromalveolate hypothesis' [1,13], which suggests that secondary plastids of Chromalveolata have been acquired through a single secondary endosymbiotic event. The most likely explanation is that the ancestor of the euglenida host cells acquired a *gnd *gene via ancient LGT from the stramenopile lineage before their divergence. This also explains well why Euglenida and Heterolobosea are robustly separated in the *gnd *phylogeny (Fig. 1) despite the close relatedness of these two lineages based on SSU rRNA gene phylogeny [35] and multiple nuclear-encoded protein phylogenies [36,37].

### Evolutionary history of *gnd *genes and plastid-lacking excavate genomes

Although our *gnd *tree topology appears unexpected compared with the prevailing view of plastid evolution [38], several gene phylogenies that suggested imprints of gene transfer between Euglenida and Stramenopiles have been reported. In the plastid-targeted phosphoribulokinase (*PRK*) gene phylogeny [39], red algal genes were basal in the eukaryotic clade and were separated from chromalveolate and green plant genes. Furthermore, euglenid and chromalveolate *PRK *genes were monophyletic and sister to green plants, and the authors reasoned that these secondary phototrophs might acquire *PRK *genes via independent LGT events. As discussed above, it is likely that Euglenida has acquired a cyanobacterial *gnd *gene from the ancestor of Stramenopiles via LGT. Although *PRK *genes are found only in photosynthetic organisms (cyanobacteria, algae and land plants) and the origin of euglenid *PRK *genes was phylogenetically unresolved, one can argue that *PRK *and cyanobacterial *gnd *genes might have gone through similar evolutionary histories. A phylogenetic analysis of plastid-targeted fructose-1,6-bisphosphatase (*FBP*) genes illustrated another case of LGT between Euglenida and Chromalveolata [40]. Thus these genes might have been transferred from the stramenopile lineage to the euglenid lineage via multiple LGT events, perhaps phagocytosis of secondary phototrophs by a phagotrophic ancestor as suggested in the chlorarachniophyte *Bigelowiella natans *[41].

**Figure 2:**
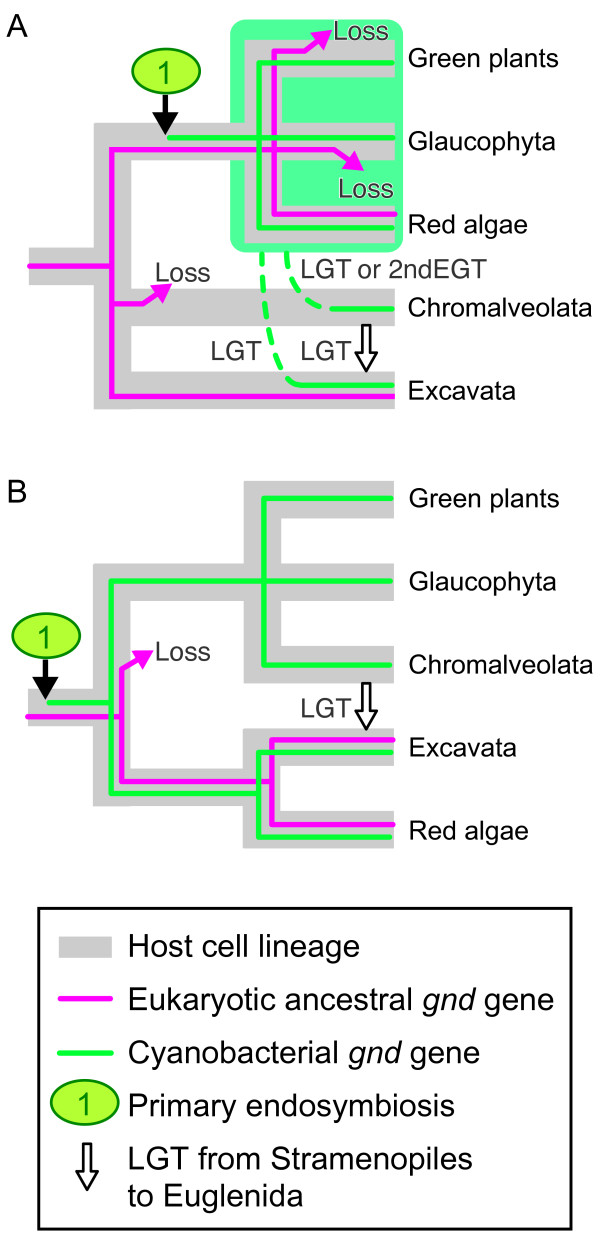
**Evolutionary scenarios on the cyanobacterial and eukaryotic ancestral *gnd *gene distribution in bikonts**. A, Traditional view of host cell phylogeny of bikonts [e.g. 45], assuming the multiple loss events of eukaryotic ancestral genes and at least two lateral gene transfer events (LGT) of cyanobacterial genes (broken lines plus white arrows). B, Alternative phylogeny [e.g. 19], assuming a single loss and a single lateral gene transfer event. Although only either the cyanobacterial or eukaryotic ancestral gene was found in Excavata in this study, only one is illustrated for clarity. Rhizaria is not shown since no *gnd *genes have been found in this lineage. 2nd EGT, secondary endosymbiotic gene transfer.

In the cyanobacterial *gnd *gene subtree, the red algal clade was at the basal position and was moderately separated from green plants and Glaucophyta. An additional phylogenetic analysis excluding Heterolobosea recovered the basal position of red algae in this subtree, suggesting that long branch attraction or artificial misplacement of red algae by heterolobosean sequences was unlikely (additional file 1). Additionally, provided that the cyanobacterial genes from bikonts were robustly monophyletic (Fig. 1), in contrast to well-characterized examples of prokaryote-to-eukaryote LGTs [42-44], it is unlikely that the cyanobacterial *gnd *genes from bikonts had been acquired via multiple LGTs from cyanobacteria to eukaryotes. Recently, two competing hypotheses on Archaeplastida phylogeny were proposed (monophyly vs. non-monophyly) [19,45]. The phylogenetic position of red algae in Fig. 1 is inconsistent with the monophyletic hypothesis of the Archaeplastida [45] unless multiple eukaryote-to-eukaryote LGTs are hypothesized (Fig. 2A). Although red algal and glaucophyte ancestries of the heterolobosean genes were not significantly dismissed, AU tests rejected the possible secondary EGT from green alga to Heterolobosea (Table 2). Hence, the eukaryote-to-eukaryote LGTs shown in Fig. 2A are likely sources of *gnd *genes in plastid-lacking protists, in terms of the monophyletic hypothesis of the Archaeplastida [45-47]. However, monophyly of red algae plus Stramenopiles (plus Euglenida) was not rejected in our statistical tests (Additional file 4), suggesting that the stramenopile genes might be attributed to secondary EGT of red alga. On the other hand, an increasing number of multigene phylogenies showed that monophyly of Archaeplastida had limited or no support [19,47-49]. Therefore it is advisable to discuss the evolutionary history of *gnd *genes, taking a different point of view on the plastid evolution into consideration (Fig. 2B). In terms of the non-monophyly hypothesis of the Archaeplastida, it is reasonable to suggest that the *gnd *gene phylogeny may reflect the host cell phylogeny as recently resolved by a multiple slowly evolving nuclear gene phylogeny [19], which demonstrated the non-monophyly of Archaeplastida and the most basal positioning of red algae plus Excavata within the bikonts (Fig. 2B).

### Possible evolutionary scenarios of plastid and host nuclear genomes

We propose evolutionary scenarios in which the common ancestor of eukaryotes possessed a eubacteria-derived eukaryotic ancestral *gnd *gene, and the bikonts lineage additionally acquired the cyanobacterial *gnd *gene via a single primary endosymbiosis [50-52] (but see [53,54] for alternative views), and then diversified into Archaeplastida, Chromalveolata, Excavata (and Rhizaria) (Fig. 2). Given that recent large-scale molecular phylogenies demonstrated the monophyly of bikonts [19,45-47] based on the rooting of eukaryotes [34], and no data providing evidence on primary and secondary plastids in the unikonts has been shown, we illustrated two likely scenarios in Fig. 2. In scenario A, we assumed monophyly of Archaeplastida [e.g. [45]], and accordingly, at least two gains of cyanobacterial *gnd *genes via LGT and multiple losses of eukaryotic ancestral genes in separate lineages of bikonts. In scenario B, we presumed that all the bikonts including secondary phototrophs and plastid-lacking bikonts had at one time acquired the primary plastid [19]. Green plants, Glaucophyta and Chromalveolata then lost the eukaryotic ancestral *gnd *gene, red algae retained both, and Excavata lost either one. In the ancestors of Excavata, loss of primary photosynthetic plastids might have triggered concurrent gene loss of either cyanobacterial or eukaryotic ancestral *gnd*. Only a single LGT event from Stramenopiles into Euglenida is considered in scenario B. Although both scenarios are compatible with our phylogenetic analysis and statistical tests, we reason that scenario B is parsimonious and more likely to explain the evolutionary history of the *gnd *genes in that less LGT events need to be presupposed. Broader sampling from various eukaryotic groups (especially in Chromalveolata and Rhizaria) will be critical to devise a more reliable evolutionary history of eukaryotic *gnd *genes, and host lineages [49]. It is also important to note that concatenated nuclear gene phylogeny of eukaryotic (host cell) lineages and data mining for cyanobacterial genes in plastid-lacking protists are supposed to be independent approaches for exploring the origin of plants. Future research will be focused on how deeply primary endosymbiosis is rooted within the bikonts, and which lineage could experience primary endosymbiosis early in the evolution of bikonts.

## Conclusion

Our present study demonstrates that (1) free-living Excavata possess either cyanobacterial or eukaryotic ancestral *gnd *genes, (2) it is statistically unlikely that heterolobosean *gnd *genes were acquired via ancient secondary EGT of green alga, and (3) Euglenida and Stramenopiles are robustly monophyletic. Although the sister relationship of this monophyletic group to any Archaeplastida lineage is not rejected by the statistical tests (Additional file 4), it is moderately separated from red algae (Fig. 1), suggesting that the *gnd *genes in Stramenopiles are not of secondary endosymbiont origin. One explanation is that a unique primary EGT of cyanobacterial *gnd *genes into Archaeplastida was followed by independent eukaryote-to-eukaryote LGTs into Stramenopiles and Heterolobosea, and then by an additional LGT from Stramenopiles into Euglenida (Fig. 2A). Alternatively, our results favor an evolutionary scenario that the *gnd *gene phylogeny reflects host cell phylogeny, and that the common ancestor of bikonts has acquired cyanobacterial *gnd *genes via primary endosymbiotic gene transfer early in eukaryotic evolution (Fig. 2B).

## Authors' contributions

SM participated in the design of the study and coordination, carried out the molecular phylogenetic and statistical studies, and drafted the manuscript. KM participated in the phylogenetic and statistical studies. MI and MW participated in the cDNA library construction and sequence analysis. HN conceived of the study, and participated in its design and helped to draft the manuscript. All authors read and approved the final manuscript.

## Supplementary Material

Additional file 1 Figure 3. **MrBayes consensus tree of *gnd *genes, constructed with 437 amino acid sites from 61 taxa**. See text and Fig. 1 for additional notes. Click here for file

Additional file 2 Figure 4. **MrBayes consensus tree of *gnd *genes, constructed with 437 amino acid sites from 72 taxa**. See text and Fig. 1 for additional notes. Accession numbers for sequences shown are as follows: *Giardia lamblia*, XP_001704443; *Trichomonas vaginalis*, XP_001298645; *Leishmania major*, XP_843439; *Trypanosoma brucei*, XP_827463;*Plasmodium falciparum*, XP_001348694; *Babesia bovis*, XP_001610335; *Theileria annulata*, XP_954525; and *Theileria parva*, XP_765720. Gene ID to *Toxoplasma gondii *gene is 49.m00043 at ToxoDB [55]. Click here for file

Additional file 3 Figure 5. **A region of the amino acid sequence alignment encompassing the EW signature of *gnd *genes**. See text, Table 1 and Fig. 4 for additional notes. Click here for file

Additional file 4 Table 3. **Comparison of alternative tree topologies by exhaustive maximum likelihood (ML) analysis**. All possible 105 trees are shown. See text and Table 2 for additional notes. Click here for file
