# Supplementary material for: Origins of a cyanobacterial 6-phosphogluconate dehydrogenase in plastid-lacking eukaryotes
Source: BMC Evol Biol. 2008 May 17;8:151. doi: 10.1186/1471-2148-8-151 (PMC2416651; doi:10.1186/1471-2148-8-151)
Supplement: Additional file 1 — Figure 3. MrBayes consensus tree of gnd genes, constructed with 437 amino acid sites from 61 taxa. See text and Fig. 1 for additional notes. [file 1471-2148-8-151-S1.pdf]

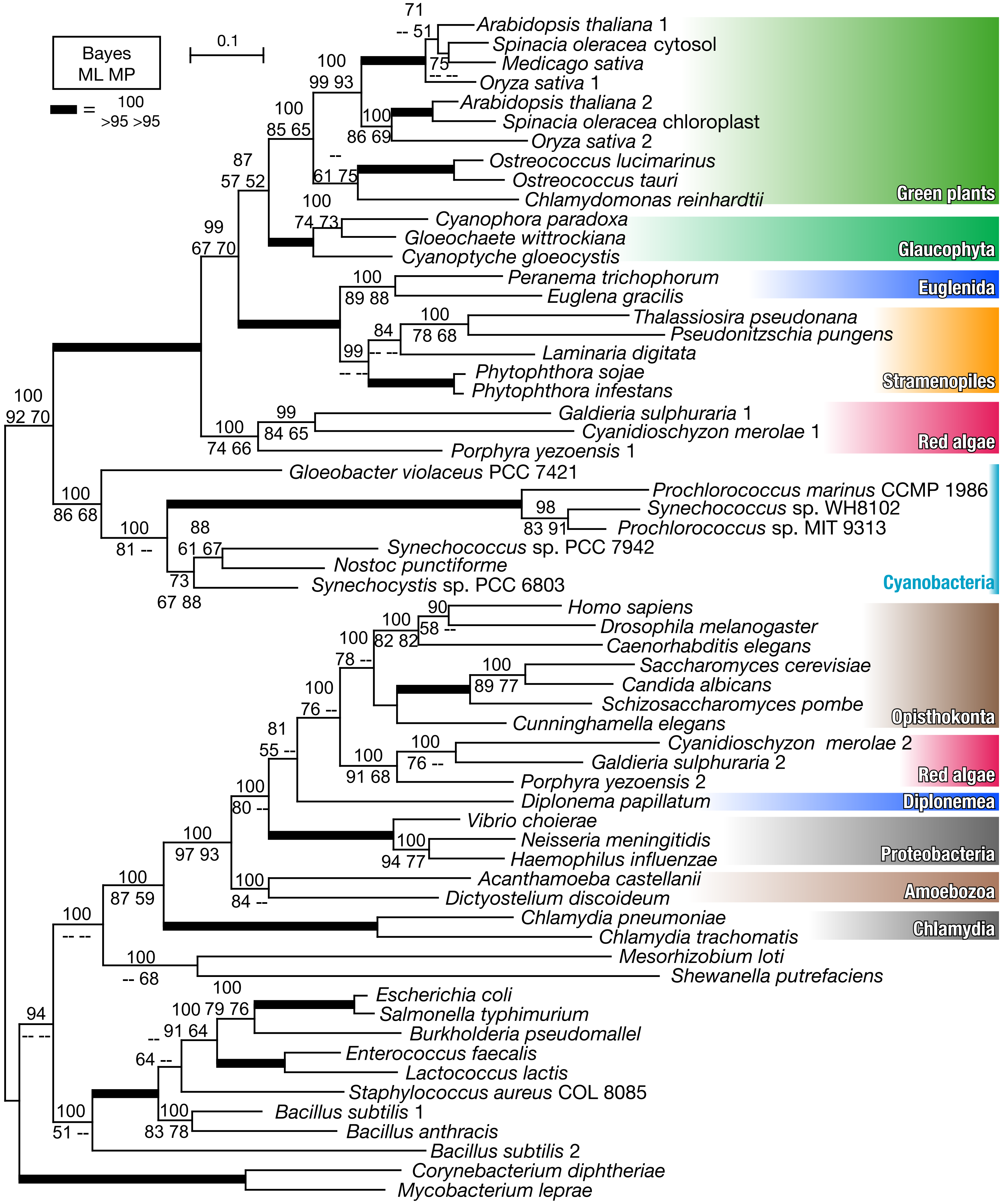

Additional file 1

Figure 3. MrBayes consensus tree of gnd genes, constructed with 437 amino acid sites from 61 taxa.

Maruyama et al. **Origins of a cyanobacterial 6-phosphogluconate dehydrogenase in plastid-lacking eukaryotes**
