# Supplementary material for: Origins of a cyanobacterial 6-phosphogluconate dehydrogenase in plastid-lacking eukaryotes
Source: BMC Evol Biol. 2008 May 17;8:151. doi: 10.1186/1471-2148-8-151 (PMC2416651; doi:10.1186/1471-2148-8-151)
Supplement: Additional file 3 — Figure 5. A region of the amino acid sequence alignment encompassing the EW signature of gnd genes. See text, Table 1 and Fig. 4 for additional notes. [file 1471-2148-8-151-S3.pdf]

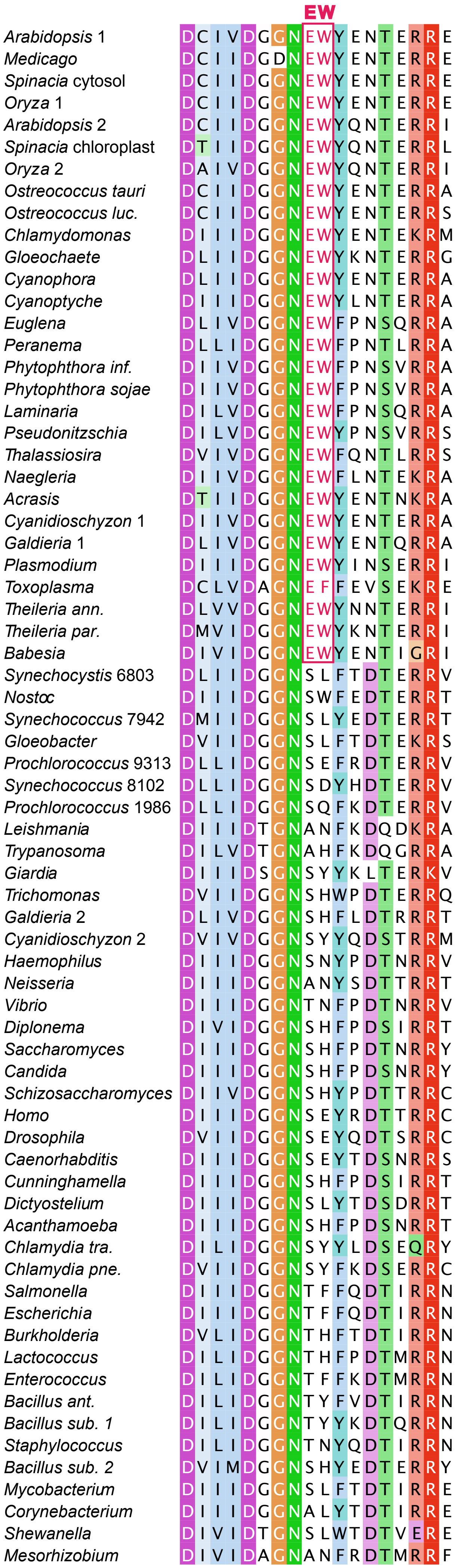

Additional file 3
Figure 5. A region of the amino acid sequence alignment encompassing the EW signature of gnd genes.
Maruyama et al. **Origins of a cyanobacterial 6-phosphogluconate dehydrogenase in plastid-lacking eukaryotes**
