# Supplementary material for: Origins of a cyanobacterial 6-phosphogluconate dehydrogenase in plastid-lacking eukaryotes
Source: BMC Evol Biol. 2008 May 17;8:151. doi: 10.1186/1471-2148-8-151 (PMC2416651; doi:10.1186/1471-2148-8-151)
Supplement: Additional file 2 — Figure 4. MrBayes consensus tree of gnd genes, constructed with 437 amino acid sites from 72 taxa. See text and Fig. 1 for additional notes. Accession numbers for sequences shown are as follows: Giardia lamblia, XP_001704443; Trichomonas vaginalis, XP_001298645; Leishmania major, XP_843439; Trypanosoma brucei, XP_827463;Plasmodium falciparum, XP_001348694; Babesia bovis, XP_001610335; Theileria annulata, XP_954525; and Theileria parva, XP_765720. Gene ID to Toxoplasma gondii gene is 49.m00043 at ToxoDB [55]. [file 1471-2148-8-151-S2.pdf]

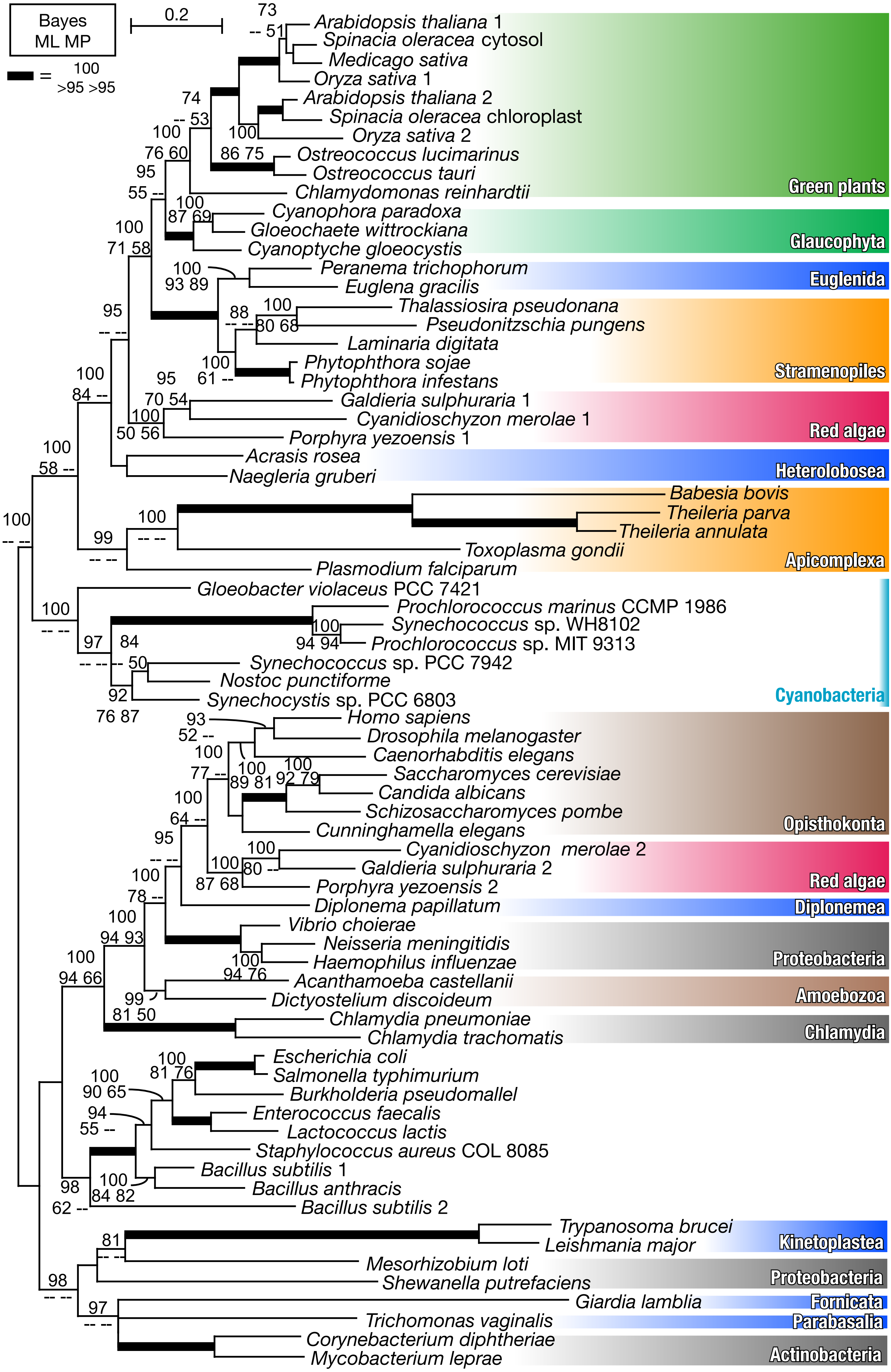


Additional file 2

Figure 4. MrBayes consensus tree of gnd genes, constructed with 437 amino acid sites from 72 taxa.

Maruyama et al. **Origins of a cyanobacterial 6-phosphogluconate dehydrogenase in plastid-lacking eukaryotes**
